# Seeing both sides: detailing the experiences of Black women mental healthcare workers serving Black youth

**DOI:** 10.1186/s12913-025-13250-2

**Published:** 2025-10-10

**Authors:** Tiyondah Fante-Coleman, Shekina Plowman, Muna Chira, Melissa Booker, Tassia Gabbidon

**Affiliations:** 1 Black Health Alliance, 112-192 Spadina Avenue, Toronto, ON M5T 2C2 Canada; 2 Social and Behavioural Health Sciences, Dalla Lana School of Public Health, University of Toronto, 155 College Street, Toronto, ON M5T 3M7 Canada; 3 Centre for Addictions and Mental Health (CAMH), 1051 Queen Street West, Toronto, ON M6J 1H4 Canada; 4 Faculty of Health Sciences, Queen’s University, 18 Stuart Street, Kingston, ON K7L 2V5 Canada; 5 Human Rights and Equity Office, Brock University, St. Catherine’s, ON L2S 3A1 Canada

**Keywords:** Mental Health, Organizations, Canada, African, Caribbean, Black women

## Abstract

**Background:**

The impacts of racial discrimination experienced by Black people in the United States and Canada have received a renewed focus. Within the mental healthcare sector, there has been a coinciding focus on how racism within and outside of mental healthcare organizations affects the mental health and wellbeing of Black youth. However, little attention has focused on the experiences of the mental healthcare workers best equipped to care for them: Black mental healthcare workers, in particular, women. Thus, the aim of this article is to 1) introduce and define Black Sistahood in Canada, 2) elucidate how anti-Black racism, sexism and other forms of oppression affect Black women, and 3) draw links to implications for the sector and the care of Black youth.

**Methods:**

Focus groups (*n* = 7) with mental health service providers (*n* = 35) in six regions in the province of Ontario, Canada: The Greater Toronto Area (GTA), Ottawa, Hamilton, Kitchener-Waterloo, London, and Windsor. Focusing on participants who self-identified as Black or mixed-race (*n* = 24).

**Results:**

Racism in the workplace created challenges for Black women mental healthcare workers. Three themes emerged: 1) Black women mental healthcare workers are subject to a toxic work environment, 2) Black women taking on multiple roles and 3) Impact on the profession and Black youth.

**Conclusions:**

Racism and discrimination impact the career trajectories of Black women employed in the mental healthcare sector. Due to this, Black women continue to leave the profession, which has a deleterious impact on the sector and care of Black youth.

**Supplementary Information:**

The online version contains supplementary material available at 10.1186/s12913-025-13250-2.

## Public significance statement

Recently, more attention has focused on the needs of Black children and youth seeking care in Canada's mental healthcare system. However, very little is known about Black women mental healthcare workers who face similar challenges and systemic barriers, namely sexism and racism. Findings showed that Black women in the mental health sector faced racism, discrimination, and sexism, which impacted their career progression, health, and wellbeing and, ultimately, the care they could provide to Black youth.

## Introduction

In the wake of the death of George Floyd and the social unrest that followed, there has been growing attention to racial injustice and the impacts of racial discrimination on Black people in the United States and Canada. A simultaneous coinciding focus was racism’s effect on the mental health and wellbeing of Black people within the mental healthcare sector [1]. Indeed, mental healthcare organizations in Canada have recognized the need to improve care provision for Black populations, including Black youth. While many multi-pronged interventions are needed within mental healthcare organizations to improve the care of Black youth, two common solutions are providing culturally responsive care and racially concordant mental healthcare providers [1]. In this study, culturally responsive care refers to mental health care that considers youth’s intersecting needs, cultural notions of wellness, experiences of anti-Black racism (ABR), and the resultant racial trauma [2].

While not every service user desires a racially concordant provider, previous research has highlighted that there is demand [3]. Reasons given for the desire for racially concordant care include Black mental healthcare providers having a shared understanding of ABR, Black service users feeling less vulnerable to racism and discrimination and increased trust and cultural responsiveness [1]. Black youth in Canada have noted that the fear of racism and discrimination from providers in Canada is sufficient enough to prevent them from seeking care [2].

 Black mental healthcare workers are often viewed by their communities as essential forms of support [3], because they inhabit a unique position within the sector as professionals and outside the profession as members of the Black community. Thus, they serve as a bridge to a community that is understandably wary of mental healthcare systems, [4] considering its history of harm and exploitation [5].Although there is a need for Black service providers to address ABR within mental healthcare, there is an equal necessity to consider the experiences of Black workers in the workplace

Unfortunately, Black mental healthcare professionals have had to navigate a difficult employment landscape within the healthcare sector that is shaped by their own experiences with systemic inequities and oppression. Indeed, the same lived experiences that are beneficial to the care and understanding of Black youth and other service users, exposes Black mental healthcare workers to potential harm. Black mental healthcare workers in Canada often find themselves grappling not only with the psychological impact of racial trauma on their clients but also with their own experiences in a predominantly white-dominated field. There is a need to understand and recognize the unique challenges Black mental healthcare workers face in a sector that is rooted in ABR and colonialism [5–7]. Specifically, it is necessary to explore the tensions experienced by Black women mental health care (BWMH) workers who provide care while simultaneously experiencing the intersectional reality of racism and sexism, deemed *misogynoir* [8]. It is necessary to do so at a time when Black youth are experiencing a period of increased crisis as it relates to mental health and addictions. A crisis fueled by a system that is poorly equipped to provide adequate mental health and addiction support to youth [9].

In this article, the perspectives of participants respond to a unique time for Black people across the globe, the spring of 2020, during the Covid-19 pandemic and a racial reckoning that began with the murder of George Floyd, which spanned countries, social spheres and sectors [10]. Their unique viewpoints and embodied experiences are worthy of themselves, but they also shed light on a system that has historically been harmful to young Black service users [3]. Understanding these experiences is necessary to ensure that there is an adequate workforce to support the needs of Black youth and to ensure a safe working environment for all mental healthcare workers.

Thus, this paper aims to centre the experiences of BWMH workers in Canada. Research questions are:

1. What is the current working environment for BWMH workers in Ontario, Canada?
  - Sub-question: How are BWMH workers positioned in these environments?
2. How does the profession's current state impact BWMH workers and Black youth?

The article begins by introducing a theory through which we can understand the experiences of BWMH workers in Canada, Black Sistahood in Canada, which draws on Intersectionality and Africanist Sistahood in Great Britain [11, 12]. We then draw on the results from a larger study focused on the mental healthcare-seeking experiences of Black youth in southern Ontario, centring the experiences of BWMH workers. We conclude with a discussion of these experiences including implications, suggestions, and recommendations.

## Method

The findings of this article are drawn from data collected for the Pathways to Care (PTC) project, a systems-change research project led by Black Health Alliance [13, 14], which aimed to increase access to mental healthcare for Black children and youth in the province of Ontario. In this article, we explore the employment experiences of Black, predominantly women mental healthcare workers working in organizations that provide mental healthcare to youth. We situate this study within a feminist epistemology that draws from Intersectionality and Africanist Sistahood in Great Britain (ASGB) [12] to speak directly to the experiences of Black service providers in Canada.

### Black Sistahood in Canada (BSC)

This article invokes a 'new' theory, Black Sistahood in Canada (BSC), which draws upon an Africanist Sistahood in Great Britain [12] and Intersectionality [11, 15], but which has been adapted for the unique contexts of Blackness in Canada. The experiences of Black Canadians have similarities to those of Black people living in the United Kingdom (UK) and the United States (US), where ASGB and Intersectionality were first defined, respectively, but also experience key differences in geography in the case of the UK, and political organization within the US. Moreover, Canada has a unique history with respect to anti-Blackness, colonialism and institutional racism that warrants its own theoretical rooting.

### Africanist Sistahood in Great Britain (ASGB) and intersectionality

ASGB confronts traditional feminist frameworks and is tied to Black feminist social justice work [12]. In ASGB, the term 'Africanist' refers to Afrocentric principles, and the theory adopts a Pan-African perspective in seeking to make diasporic ties. ASGB references Black people's direct ancestral ties to Africa in Great Britain. 'Sista' captures the centrality of Black womanhood and the importance of lived experience.'Sista' refers to a positive relationship with Black womanhood [12].'-hood' references areas where Black women's identities align and diverge, linking those differences and similarities across the diaspora. In sum, ASGB is rooted in finding commonalities across the diverse Black diaspora, including with Black men, and prioritizes creating a separate paradigm that adequately explains the experiences of Blackness within their context. Moreover, ASGB speaks to the experiences of Black women in Great Britain, making a clear distinction from the embodied experience of 'Britishness,' instead the theory speaks to the UK's specific history regarding 'race, ethnicity and racism' rather than drawing from other contexts [12].

As ASGB is rooted in a British context, intersectionality is similarly rooted in a US context [15]. Moreover, like ASGB, intersectionality is rooted in a feminist epistemology and a social justice orientation. Intersectionality focuses on structural inequalities and attends to how systemic power is wielded over individuals [15]. intersectionality lends space to discussions about multiple dimensions of oppression and how oppression is located within a larger socio-political context. In this article, we are interested in how racism intersects with the professional experiences of Black mental healthcare workers who care for Black youth.

#### Black Sistahood in Canada

In this study, we aim to foreground the experiences of Black women in Canada, think about the multiplicity of their experiences, and link those experiences to the systems of oppression related to race and gender. Thus, we use BSC to signal this study's roots in social justice, leveraging the study's findings to drive change. BSC draws from ASGB's focus on making the'invisible'visible and questioning when hypervisibility is used to oppress Black people further [12].'In Canada' pertains to the unique experiences of racism in Canada, which does not have the drive to specific innocence or unfamiliarity as it does in the UK [16], but also does not have the same deepness of institutionalization that is spoken about in the US [17]. Although intersectionality encompasses gender identity, it focuses on unique forms of oppression and structural inequalities rooted in marginalized identities. In contrast, BSC builds on intersectionality by explaining what experiences of Blackness and ABR in Canada look like for women across the diaspora. BSC contextualizes intersectionality by underscoring Canada’s unique experiences of institutional ABR and colonialism, and the tendency to adopt a colour-blind approach to addressing ABR across the healthcare system, including mental healthcare institutions.

While Canada did not have codified laws such as Jim Crow, legal and other institutions in the country have and continue to discriminate against Black people in Canada. Examples include Prime Minister Wilfrid Laurier's remarks about people of African descent not being welcome in the British Colony that became Canada in 1911 [18], to the legacy of Africville, segregated schools and the denial of Black students to medical schools in the country [19]. Black Canadians continue to experience racial discrimination, as evidenced by a recent report that police forces continue racially discriminatory practices such as carding, post-George Floyd [20].

Returning to the theory, BSC does not speak necessarily to citizenship because some Black people residing in Canada may not feel, or be legally regarded as, Canadian (e.g., temporary foreign workers), but still experience anti-Blackness and remain embedded in the larger diaspora. At the same time, BSC speaks to the diasporas in the UK whose histories mirror those in Canada [16]. Canada and the UK are commonwealth countries, with newer generations moving from countries colonized by the British Empire. The ethnic makeup of most of their Black populations, typically from countries in Africa and the Caribbean, are similar due to the British colonial history of these regions and differ slightly from the experiences of African Americans in the US. Moreover, the experience of the simultaneous (though often reluctant) celebration (e.g., Caribbean Carnivals) [21, 22] and denigration (e.g., Windrush scandal and deportations) [23] of Caribbean populations, are also similar.

### Project and research design overview

The findings of this article are drawn from the PTC project, a community-based participatory research project; detailed information can be found in Fante-Coleman, Jackson-Best et al. [1]. In this article, we leverage 23 qualitative focus groups conducted across the province of Ontario focused on Black youth's mental health and wellbeing and ability to access mental healthcare. The project engaged Black youth, family and caregivers and service providers, and the latter group's perspectives form the basis of this article. The original study followed a qualitative approach using thematic analysis [24]. It became increasingly clear during data analysis that exploring the perspectives of Black mental healthcare workers was necessary, though it was not the intention of the original study [1]. Thus, in this article, the authors reflexively revisit Black mental healthcare workers' experiences [25] through a lens informed by Black Sistahood in Canada derived from ASGB [12] and intersectionality [15].

The project employs a critical qualitative research approach that is rooted in the principles of community-based participatory research, as this was the basis of the original study. However, for this article, no one specific methodology was leveraged. Instead, best practices across methodologies were utilized to ensure that the methods were congruent with the goals of this article [26], which was to identify the experiences and challenges faced by BWMH workers in Ontario. This is in part because the project draws on numerous theoretical orientations, including feminist epistemologies, as BSC is informed by intersectionality and ASGB [1, 12, 15], and community-based participatory approaches [27].

Working across theories and methodologies has become increasingly accepted in critical qualitative research as described by Tracy [26], which uses eight criteria to determine qualitative rigour: worthy topic, rich rigor, sincerity, creditability, resonance, significance of contribution, ethics and meaningful coherence. In this article, the subject is framed and analyzed through the lens of feminist approaches which require an attentiveness to structures of power and a focus on reflexivity [28]. In addition, the inclusion of co-authors who identify as Black women was rooted in principles of community-based research [27]. The use of a historical analysis to understand the racial component of BWMH workers’ experiences is influenced in part by BSC’s rootedness in a focus on the diasporic history of Canada. This analysis is bolstered by devices that contribute the generation of new data [25], as the authors acknowledge that the results of this article differ from the original intended purpose of the overall project.

### Researcher description and reflexivity

The authors of this article collaborated with a multidisciplinary team consisting of researchers and health promotion specialists from the PTC project, students and practicing mental healthcare workers. All authors identify as Black women. We bring to this work our professional and personal experiences, subject matter expertise and research knowledge. As a team, we met multiple times over a year to engage in a collaborative review of the data collected and results.

#### Reflexivity

Reflexivity was integral to our interpretation of the results and the presentation of the data [15]. During data analysis, our standpoint was considered, and we noted our intention to promote fairness in the workplace for all mental healthcare workers, particularly Black women. In doing so, we reflected as a team on what we 'knew' and how we claimed it to be known, seeking to emphasize the experiences of the participants we spoke to [25]. We wanted to uncover the experiences of Black people in these professions in Canada because we felt that they have not yet been spoken to as it pertains to racism. By going back into the data, we sought to explore how BWMH workers experienced racism in mental health in their respective organizations and how that was unique to the specific historical, cultural, and social context of Canada. We wanted to know how they described their experiences and what meaning they made of those experiences.

We are a team comprised of women, in alignment with most caring professions [29]. The original study was about access to care for Black youth, however, in this article we intentionally sought to explore how power and oppression affected mental health workers and, in turn, Black youth. Thus, we produce an account that we feel best reflects participants' experiences while recognizing that our experience impacts the interpretation of the data. Naming these objectives is intentional as we recognize that eliminating 'bias'[25] is not the objective of qualitative work, and we reframe our subjectivities as a benefit to this analysis because we are situated in and embody the same worlds as our participants [30].

#### Participants

The PTC project engaged 128 participants, including Black youth (*n* = 66), family and caregivers (n = 27), and service providers (n = 35). This article explores the experiences and perspectives of the service providers who self-identified as Black or Black and mixed race (*n* = 24, 57.1%), although the wider sample included participants who identified as people of colour (n = 9, 21.4%) and white (*n* = 5, 11.9%) (see Table 1.). Participants were 27–54 (M = 37.36, SD = 7.868) years of age. Participants were able to self-identify with more than one ethnicity. Most participants self-identified as Caribbean (*n* = 18, 51.4%), North American (*n* = 8, 22.9%), West African (n = 5, 14.3%), (see Table 2.). Four participants did not respond. Most service providers identified as women (*n* = 28, 80%), while three (8.6%) identified as men. Black women (*n* = 22) represented 62.9% of the sample. Complete demographic information can be found in Fante-Coleman et al. [31].

**Table 1:** Race of Service Providers

**Race**	Service Providers (*n* = 35)	Percent (%)
Black	24	57.1
Person of Colour	9	21.4
Indigenous	0	0
White	5	11.9
No Response	4	9.5
Total	42	100.0

**Table 2:** Ethnicity of Service Providers

**Ethnicity**	Service Providers (*n* = 35)	Percent (%)
Arab	1	2.0
East African	3	6.0
West African	5	10.0
Southern African	3	6.0
Caribbean	18	36.0
Eastern European	0	0
Northern European	1	2.0
Southern European	1	2.0
Western European	4	8.0
North American	8	16.0
East Asian	1	2.0
Southeast Asian	1	2.0
Indigenous	0	0
No Response	4	8.0
Total	50	100.0

### Participant recruitment

Participants were recruited using purposive sampling via email, community liaisons and social media calls [1, 32]. We recruited participants through engagement with community and social service organizations. Participants were asked to participate in focus group sessions to discuss the experiences of Black children and youth in the mental healthcare system. While the focus of the PTC project was the experiences of Black youth, service providers were not required to self-identify as Black; however, they must have 1) worked as a mental health professional (e.g., therapist, social workers, nurse) in Ontario, 2) had worked with Black youth and 3) be over the age of 18.

Before the focus groups, TFC called participants and conducted a screening call to ensure participants understood the purpose of the study. Participants were given an informed consent form and a demographic survey to complete after the discussion. Informed consent was obtained from all study participants.

### Data collection

Focus groups (*n* = 7) took place between May 2020 and August 2021 in six regions in the province of Ontario: The Greater Toronto Area (GTA), Ottawa, Hamilton, Kitchener-Waterloo, London, and Windsor. Two focus groups took place in the GTA. Sessions lasted approximately two hours and were conducted via Zoom in English by TFC, an experienced qualitative researcher. A focus group question guide was developed for this study. Questions were semi-structured, co-developed with members of PTC’s Youth Action Committee and focused on participants' perceptions of Black youth's access to care, their own experiences in the mental healthcare care system, and suggestions for improving care. For the overall study, saturation was met when focus groups were completed in all regions and no new information was gleaned. Data were returned to participants for member checking to ensure they reflected participants' viewpoints. All participants were given 40 CAD as compensation via electronic transfer or cheque. PTC project activities received ethical approval from the Community Research Ethics Office (project #154) and were completed in accordance with the principles of the Tri-Council Policy Statement [33].

### Data analysis

Original data analysis was conducted using thematic analysis, following the methods outlined by Braun & Clarke [24]. Original coding was completed by PTC researcher (TFC), who has extensive experience in qualitative research. For this article, the research team revisited and reanalyzed the initial codes through the lens of exploring the experiences of Black mental healthcare workers. The project team met biweekly to discuss the theoretical orientation of our project, outlining the use of the theory we intended to leverage and discuss our reflexivity as a team. We then revisited and reviewed the codes to determine their relevance to the topic of interest and coherence with participants' overall narratives. We aimed to 'open up' the data by combining our multiple knowledges with other research. This process is similar to the process defined in ASGB, where we reconsidered and reconceptualized the data [12], making a place for a new concept. Thus, codes were designed to meet the analytic scheme for Black Sistahood in Canada, interpreting analytic strategies from ASGB and intersectionality [12, 34].

When reviewing the data, we looked for guidance from Eakin and Gladstone's [25] concept of 'value-adding' to the research, where we used ourselves to analyze the data. We focused on the standpoint of BWMH workers, thinking as analysis for contextualization, creativity and interpretation, and critical inquiry. Analysis was considered complete when the authorship team felt that there was cohesiveness to the'story'of the themes and that participant reflections were accurately represented.

## Results

ABR^1^ and sexism in the workplace created challenges for BWMH workers at the systemic, organizational, and individual levels. Three themes emerged: 1) *Workplaces are unsafe for Black women* and, 2) *Black Women Taking on Multiple Roles and 3) Impact on the Profession. 1) Workplaces are Unsafe for Black Women* had one subtheme: *Racist Workplace Policies and Black Women Advocating for Accountability.* The theme *Black Women Taking on Multiple Roles* has three subthemes: *Black Women Filling in the Gaps, Visible DEI Leaders, or Are They?* And *Doing the Hard Work, then Exiting.* The final theme *Impact on the Profession* had also had three subthemes: *Recognizing the Mental Healthcare System as Discriminatory, Deciding to Leave the Profession* and *Finding and Giving Support as a Form of Resistance.*

### Workplaces are unsafe for black women

Mental healthcare is often observed as feminized work, and when this intersects with racism, it results in gendered experiences of toxicity and discrimination. For example, cases of sexual violence and harassment occur in the workplace. One participant noted that she was subjected to a professional environment where her complaints about a co-worker’s racist comments were disregarded:

So even the higher-ups come to me like, 'oh well…Do you think that it could have been a misunderstanding?' or ' do you think that he was just interested in you?'...But I definitely felt a lot of the bricks, and I felt that it was racial. - [Lana, FG9, Ottawa]

Lana's experience echoed that of another participant, Robyn, who faced sexual harassment by a co-worker and recounted that her organization's management attempted to diminish the severity of her ordeal. The sexual harassment Robyn experienced bore significant racial overtones. It exacerbated the sense of vulnerability she felt in the workplace:

...I believe [I] was a victim of sexual harassment, and I do think it was because I was Black. Because the sexual harassment was racially based, right? And even being on the other side and …Having to defend, like I was even asked, you know…'Could it be that that person just wanted to ask you out?'- [Robyn, FG9, Ottawa]

Not only were Black women encountering sexual harassment at work, but a racist dimension added to their harassment. This occurrence was aggravated by the inaction of their superiors, who failed to address concerns related to sexual harassment. Discomfort in the workplace also arose when only a few Black women were present in their professional environment, rendering them isolated in the face of racism.

...At times, people don't want to refer to me as a Black person. And I was like, 'I'm a Black woman.... Don't call me brown.' - [Gloria, FG12, Hamilton]

Whether intentionally or subconsciously, Gloria's coworkers disregarded her racial identity and conveyed that her Blackness was something that should not be seen or addressed, insinuating a detrimental connotation with Blackness. The participant also observed this sentiment in her community, as others dismissed her children's racial identity.

Furthermore, when Black women communicate that these spaces are unsafe for them and their clients, they are penalized instead of protected. Black women are made to feel as though they should not be speaking out. One participant explained that she was punished by superiors when she reported instances of racism in her workplace:

...And as I watch it happen, and if I comment on it, I'm the one to be reprimanded, because how dare you speak about it? We shall do this because the laws allow us to do this. So how dare you come and say something about it? - [Tania, FG18, London]

Overall, Black women experience racism and sexual harassment in the workplace, creating feelings of insecurity and discomfort at work. The lack of managerial support in response to racism further magnifies these negative feelings.

#### Racist workplace policies and black women advocating for accountability

Participants described the presence of policies in the workplace that enable racism and impacted both BWMH and their clients Specifically, BWMH did not feel protected by their organizational policies:

You know…Everywhere I've ever worked, I read their policies, and I read every one of them...But the rules, the laws, the laws allow for Black boys, Black girls to be treated like garbage. And then people are held in their positions, people are allowed to be suspended, maybe with pay, people are allowed just to continue working as usual. You know, nothing happens. And because of these laws, because of these rules, it's like, society sees the Black child as less than, so they're going to be continued treated like less than.

- [Tania, FG18, London]

When organizations uphold discriminatory policies, it reinforces the idea that Black youth are less than their White counterparts, to detrimental effect. There are little to no rules to prevent racism, and as a result, it goes unaddressed. One participant explained that she must constantly advocate for Black workers and clients in her professional environment, which takes a toll on her:

I'm trying to find the appropriate words...I think tiring would be one of the words I would use. Having to point out Black staff struggles, to being one of the few people who constantly have to advocate for Black people that come into our organization. - [Lana, FG9, Ottawa]

Participants shared that their complaints concerning racist policies or racism exhibited by coworkers were not handled appropriately. However, participants believe this can be prevented when there is accountability:

...I think the presence of policy and the accountability of making sure that they're implemented all the time is -there's a disconnect for sure…Yeah, that accountability in ensuring that across the board, no matter who is working with clients in terms of like the staff equity as well, ensuring that that translates, I think that it's due to maybe perhaps a lack of education and a lack of understanding of how that can happen. - [Mary, FG1, Toronto]

Furthermore, the consensus among participants was that there was a lack of evidence-based research on anti-racist policies, which contributed to toxic workplaces. In response, one participant pushed for more research that would facilitate creating policies designed to protect Black mental healthcare workers and their clients:

There needs to be evidence-based…practices. I think the way we collect information about people and then report it back to like larger policies needs to happen as well. I think there needs to be more care put into…creating evidence-based policies that affect the general population. - [Aisha, FG2, Toronto]

Lastly, when Black workers witnessed blatant racism from their coworkers and advocated for themselves, they experienced a lack of support from management. One participant stated that her non-Black colleagues needed to be trained to recognize discrimination:

…I think coming from like it is an individual work being done on a deeper level depending on the case worker for sure, but…It is a little bit of both. I think there just could be a little bit more training on all of the different intersectionalities…but I think there isn't anyone who will be able to fully understand them it's just kind of working with [clients] to support their own understanding of themselves... - [Belle, FG1, Toronto]

Overall, the presence of discriminatory policies in mental healthcare and the lack of institutional support perpetuated a culture in organizations that mistreats and disempowers Black youth and demoralizes Black mental healthcare workers.

### Black women taking on multiple roles

Black mental healthcare workers often discussed how their identities positioned them to take on multiple roles within their organizations. Often, this led to additional work over and beyond their regular duties and responsibilities. For example, Black women were often expected to be the main supports of Black youth in their workplaces, usually because organizations had very few support structures in place specifically for Black youth. Indeed, respondents noted that they were expected to be the 'fixers' for Black youths' needs in their workplaces but often did not receive additional support or resources for this labour. Black women respondents noted that they felt these increased expectations related to their roles in their communities, where they were expected to be both 'strong' and 'caregivers' to others.

#### Black women stepping up and filling in the gaps

A common sentiment among Black women respondents was that to ensure that Black youth received the care they deserved, they often had to fill the 'leadership gap.' This was particularly the case in the Spring 2020 because Black women felt compelled to address the harms of racism and its effects on Black youth in what felt to them was a vacuum in non-Black leadership's ability to proactively identify and respond to issues related to systemic racism within the workplace to address ABR. During this time, Black women mental health workers became *impromptu leaders* who led organization-wide changes to increase inclusivity to serve Black youth better. In these impromptu roles, Black women were hyper-visible, as they were upheld as 'mascots' for the supposed transformational changes organizations were undertaking.

However, not all this leadership was voluntary. While many respondents felt compelled to act because they found their organizations'responses lacking, others felt that they were effectively forced to do this additional labour.

I definitely find that I've either been used to try to…calm a situation down because I'm Black… If somebody who was struggling with mental health or addiction issues who is also Black, you know I was kind of like the wingman to kind of go and handle those situations - [Robin, FG12, Hamilton]

Going out of their way to assist Black service users was particularly challenging for respondents because they were often among the few Black women who worked within their organizations. Respondents also noted that they often took on an added human resources role that was not part of their job description.

My experience…was twofold. So one was, I was the only Black manager on a small team, so I was expected to address all the racial issues…Then a part of that actually led

to me now being out of a job because I got in a bit of a confrontation with the director. So basically, what happened was last spring, whenever the George Floyd killing and the uprise of the Black Lives Matter movement [was], most organizations were like actively putting out statements or doing stuff, my organization did nothing. – [Lexis, FG12, Hamilton

#### Visible DEI leaders, or are they?

Due to a lack of organizational commitment, the onus often was on respondents to identify solutions to ABR within their organizations. Unfortunately, this was often a catch-22. Despite being coerced into this additional labour, BWMH workers lacked the institutional support to enforce these changes. Participants described the proposed solutions as 'diversity checklists' that did not lead to real systemic change. For example, diversity, equity, and inclusion trainings were not implemented in conjunction with changes to practice and policy. Moreover, these trainings were often attended only by Black mental healthcare workers because their colleagues often left the burden of care for Black youth with them. When respondents pushed for broader and further reaching changes, they often faced pushback that reinforced the status quo:

I felt that it was going to be on me to ensure that there was a critical lens of what was already there. Because what ended up happening was, we went through it. Everyone said it was fine the way it was, of course. I thought there were a lot of holes, gaps, things that could be done…That ended up being a prominent experience for me, and I'm sure there are many other Black people in all the sectors who became the equity, diversity, inclusion person for free, when everyone, you know, discovered that racism and police violence existed last year. – [Sharon, FG15, Hamilton]

This participant described the challenges they faced when they tried to lead a review of current policies and practices related to ABR within their organization. These policies affected employees' experiences and best practices for caring for youth. In these moments, BWMH workers were essentially remade to be invisible, as the attempts to make changes were moot.

They have, you know, maybe a couple little things in the manual about loving each other and not judging and reporting harassment. We're all the same, that kind of BS, but it's not often acted on, and usually by senior members of the team as well…Yeah, I don't want to say stereotypes, but just even from my experience, it is often those people that seem to have hate in their heart to [people of colour]. - [Robyn, FG18, London]

Another respondent discusses how she was automatically assumed to be responsible for addressing ABR within her organization and was reprimanded for not doing so:

… the director asked around the table, it was me and two other white managers like, what's happening? Both managers are like, weell, I'm white, this went over my head, like, you know, I have no Black friends. Didn't matter to me, like just basically went over my head.'So, she said,'okay.' I said,'Well, I've never seen the organization being active in anything like this. They've never taken a stance. So…I have my business outside of work and she went on my website…and she said to me, 'you know what, Lexis, this is all your fault. This is your lack of leadership why this wasn't addressed.' I said, no, it's not. That's not in the scope of my work. This wasn't something you asked me to address.

I'm not going to take the fall for it. – [Lexis, FG15, Hamilton]

Lexis continues, describing how her boss expected her to take on labour beyond her role because of the work that she did outside of the organization:

…I said to her, 'I can help you to get something together to address the staff if that's what you would like.' She said, 'no, because I didn't do anything wrong here. 'Right? She said, 'there's COVID, I did nothing wrong. 'She actually went on my business website, printed out all the services that I offer through my business and said, 'this is what, this is one of your strengths and I want you to do it.' I said, 'no, this is not the scope of my work here, the organization has a committee, so you can bring it back.' She's like, 'no, you need to lead it because this is your strength.'- [Lexis, FG15, Hamilton]

Because Lexis was recognized as a leader in this area, her boss pushed her to lead this work. However, Lexis still faced a lack of institutional support to do the work the way she wanted to:

…I said, if I'm going to do it, I'm going to do it my way, so we will need to have open conversation with the staff team about [ABR], and she said, 'no.' I said, 'you know what, I can't do diversity your way,' because she wanted me to do it as a cover-up and I can't do it your way. I can't do anti-racism training your way because she goes in denial that everything's fine. We're doing good. I said, 'I can't do that.' I was targeted from there on. I realized what happened was the other manager, I trained her [but] she wanted to leave. The director didn't want her to leave. As soon as I left, within two weeks, she promoted her to being a director to take over. Now they just kind of don't deal with the anti-racism stuff. - [Lexis, FG12, Hamilton]

#### Doing the hard work, then deciding to leave the profession

Unfortunately, Black women in mental healthcare organizations were often seen automatically as the people best suited for challenging and emotionally laborious work. Lexis from Hamilton continues:

…This was ongoing for five years, where anything race, diversity-related would fall on my plate. The staff would come to me, 'Lexis, why aren't we addressing this,' …but the expectation wasn't for all the managers, it was just me because I identify as Black. So, there was actually an unbalance in the organization where my team was fully trained in Intersectionality, whereas the outreach team never touched it because their manager wasn't doing it. And the director wasn't, it wasn't on our agenda. - [Lexis, FG12, Hamilton]

In another example, respondents noted that they experience colleagues deferring 'harder' cases and situations to Black women because they didn't want to seem like the 'bad guys' for doing their jobs.

For Black women in these roles, the expectation that they would take on these extra caregiving roles, held by themselves and others, often led to burnout because they felt that they would have little support if they voiced their concerns. Burnout is characterized as a state of emotional, physical, and mental exhaustion resulting from prolonged and excessive stress [35]. The increased expectations impacted their health and wellbeing. Over time, these factors, and their own experiences of racial and gendered oppression in the workplace led them to leave mainstream organizations and set up private practices where they could control their work environment and the care environment of the Black youth they served.

You know what I mean? We burn out because we're doing that and we're doing work that's providing us with income and for us to live and feed our families. So, it's very tiresome and taxing – the emotional tax on us, it's too much, you know. - [Katrina, FG9, Ottawa]

As one social worker from Kitchener-Waterloo recounted:

…Luckily, I had the support to start my own private practice, and when I started the private practice, that's when I said, you know, people are going to get all of me, and I'm going to give them the best of me, and hopefully, there will be a few people that look like me…So, in terms of my job now and my boss, she's a lovely lady (laughs). It's me. - [Miranda, FG15, Kitchener-Waterloo].

### Impact on the profession and black youth

Intersectionality and race constitute the primary focal points of this study. The women in this study experienced discrimination and microaggressions before entering and during their professional careers. Lack of Black representation and support led to burnout, mental health workers leaving the profession or going to Black-owned private practices in search of more fulfillment and workplace solidarity. Through finding supportive workplaces, the mental health workers showed higher dedication to providing mental health services, which divested away from mainstream mental health care and provided a higher calibre of support and respect to Black youth and communities. The following section will look deeper into these findings.

Three distinct subthemes were identified: 1) Recognizing the Mental Healthcare System as discriminatory, 2) Deciding to leave the profession, and 3) Finding and giving support as a form of resistance.

#### Recognizing the mental healthcare system as discriminatory

A single participant offered a thorough account of their encounters with unfair recruitment processes, microaggressions, and the glaring absence of Black representation. These adverse experiences provoked feelings of inadequacy and a sense of exclusion among the women:

... I'm pretty much the only Black person. So, it's pretty much white folks... I am working from home, so that has been taken off like an ease. Just imagine, like, walking into a space where, you know, it's just you every day.... There's microaggressions... that I've been going through all these years - [Gloria, FG12, Hamilton]

This narrative highlights the significant challenges faced by this respondent as the sole Black person in her workplace. She consistently encountered subtle but hurtful microaggressions. The phrase, 'it's just you every day,' reflects her isolation and the lack of a supportive workplace community that can relate to her experiences. Her preference to work from home starkly underscores the unwelcome work environment she has endured for years, emphasizing the negative impact of being one of the only Black professionals in her workplace.

The scarcity of Black representation is compounded by an observation made by another participant, who articulated the need for Black mental health professionals capable of delivering culturally responsive care and possessing a deep understanding of the pervasive racism and microaggressions that Black individuals routinely experience. This narrative serves as a reminder of the need for awareness and change, particularly among Black youth, in addressing systemic racism and microaggressions.

I thought to myself, we need to have service providers that cater to Black people and that actually have education and knowledge of what it is like to be a Black person experiencing racism in this... country that we live in. – [Halima, FG12, Hamilton]

This imperative extends beyond its significance for Black youth and communities alone; it holds equal importance for Black mental health professionals. It aims to foster an environment where Black mental health workers can find understanding and engage in open discussions about the unique challenges they face as Black women within society.

Such an environment not only promises more constructive and positive workplace experiences for mental health workers but also simultaneously enhances the quality and effectiveness of therapeutic care offered to Black youth and individuals seeking support.

Another mental health worker emphasized a critical aspect of the system and its role in discouraging Black women from entering the profession. This discouragement primarily stemmed from the widespread problem of ABR:

... There are a lot of people who would love to do therapy and would love to work specifically with the Black community, I hear that often from other people within the field who are Black. But it's very hard to get to a place where the system tells you that you're not good enough, basically, that you're not educated enough. – [Sharon, FG15, Kitchener-Waterloo]

Many participants expressed how systemic, societal, and racist influences have instilled enduring imposter syndrome and feelings of educational inadequacy—predating their entry into their respective professions and persisting throughout their careers. The insights provided by Sharon underscore the broader context of marginalization and mistreatment faced by Black women before joining the workforce. This highlights the essential need to address ABR for women entering the workforce, recognizing the Intersectionality of Black women, and dispelling misconceptions about the knowledge and education of Black individuals.

#### Deciding to leave the profession

The absence of adequate support emerged as a pivotal factor compelling numerous Black women to exit the profession. Lana, a service provider from Ottawa, offered a personal account, revealing her own experience of never receiving the necessary support within her workplace, which ultimately prompted her departure:

...A manager or someone else above our positions would be responsible but also available to debrief, you know, provide any counsellor care type things. That never happened in the one-and-a-half years I was there, and if we reached out to them and asked for that support, it felt like we were bothering them for that support... so that's why I left – [Lana, FG9, Ottawa]

Burnout, ABR, and vicarious trauma stand out as significant factors impacting the retention of Black mental health workers. As evident in the previous quote, Lana's departure was exacerbated by insufficient support in managing these challenges. Fostering a workplace environment that is both positive and supportive is paramount for retaining Black mental health workers, given that Black women frequently bear the weight of addressing these multifaceted issues independently.

There wasn't enough attention, or maybe even knowledge or recognition of the impacts of racialized people working with other racialized people's trauma... Yeah, I think that that there could have been, and should be more done – [Sharon, FG15, Kitchener-Waterloo]

Sharon is speaking to the heavy burden of vicarious trauma, particularly for Black mental health workers, who experience its emotional toll significantly. Vicarious trauma can be draining and emotionally taxing for mental health professionals. In the absence of proper support, many of these dedicated mental health workers find it necessary to take leave to prioritize self-care, and regrettably, some may even choose not to return to their roles due to the overwhelming exhaustion it entails.

#### Finding and giving support as a form of resistance

A prevalent theme among the research participants was the need to support Black youth and communities. Driven by a sense of purpose and facilitated by effective management, the BWMH workers demonstrated a deep dedication to providing culturally responsive care. They actively sought to redirect their services away from conventional mental health services and instead deliver tailored care that acknowledges and respects Black culture. Many BWMH workers worked in organizations unwilling to address ABR, so participants found other creative ways to provide mental health care for Black youth. For example, they discussed opening their private practices, leaving their roles, or leaving the field altogether.

Luckily, I had the support to start my own private practice. And when I started the private practice, that's when I said, you know, people are going to get all of me, and I'm going to give them the best of me. And hopefully, there will be a few people that look like me – [Miranda, FG15, Kitchener-Waterloo]

Miranda expresses her dedication to providing her clients the highest standard of care. Many Black mental health workers expressed that when they felt fulfilled and worked in a supportive environment, they had the energy and drive to provide excellent care to their communities and clients. As a result, this led to better health outcomes and experiences for Black youth receiving mental health care.

A strong sense of identity and representation plays a pivotal role in the mental health of youth. BWMH workers offer a unique level of comfort and care that fosters trust and a safe space for youth to express their mental health needs openly. Judy, a mental health worker from London, Ontario, touched on a critical factor needed for youth to feel safe and openly express themselves to their mental health worker:

They're [Black youth] walking into an environment where they don't see themselves, they just see a bunch of white people that are trying to be nice, but I think we have a major problem with our optics because I could go days on this floor without seeing a Black worker in any capacity – [Judy, FG18, London]

Judy provided a distinctive perspective on how youth perceive the mental health services available to them and emphasized the significance of feeling represented within these services. Following on the topic of Black representation, Ebony, a mental health worker from Windsor, touched on the importance of BWMH workers and how that impacts youth getting services:

… But even to be in a school system and see someone who looks like you. Who is providing service even if they're not providing it to you? Right, I think that makes a difference. That's part of that prevention that we're talking about, [it] is there aren't enough people who look like us who provide service. – Ebony, FG21

Ebony's account underscores the importance of having Black mental health professionals when delivering services to Black youth. Representation extends beyond offering culturally responsive care; it instills in young individuals a sense of belonging, supports the development of trustworthy therapeutic connections, and aids in implementing preventative health strategies.

## Discussion

The purpose of this study was to understand the current working environment of BWMH workers in Canada, understand their positionality in their working environment and to document the impact of their experiences on the care that Black youth receive. Study findings suggest that BWMH workers are subject to harmful professional environments which negatively impacted their psychological wellbeing. Participants encountered both racist and sexual harassment in their professional environment, as well as penalization for addressing racial disparities within their organizations. The experiences of BWMH workers in their workplaces often led to burnout or disengagement from their profession.

Several studies have noted that Black mental health professionals significantly impact the mental healthcare of Black youth, because they are better equipped to provide Black youth with culturally responsive care [1, 2, 36]. Thus, BWMH workers’ exodus from the mental health care sector impacts the quality-of-care Black youth receive, providing a rationale for a more fulsome understanding of their experiences in the workplace.

BSC provides a lens to understand how the experiences of BWMH workers in mental healthcare organizations are tied to their unique experiences of Black women in Canada, which has specific implications for their rootedness in the specific political realities of how racism and sexism are addressed within the country.

### Workplaces as unsafe for black women

Despite attaining higher socioeconomic status and education in corporate or healthcare work, Black women in our sample did not escape instances of discriminatory practices in the workplace. Black women in this sample experienced workplace violence that was tied to racialized sexual harassment. Participants reported experiencing racist comments from colleagues, as well sexual harassment that was tied directly to their Black identities. These experiences had detrimental effects on their employment and psychological wellbeing [4, 5, 37–39].

It is possible that these collective experiences could be attributed to stereotypes. For example, participants’ experiences evoke the ‘Jezebel’ stereotype, which hypersexualizes Black women as promiscuous [40]. The oversexualization of BWMH workers places them at risk of continued sexual harassment and assault in the workplace [40]. In addition, participants noted that their colleagues often dismissed the importance of race and experiences of racism for BWMH workers in their personal and professional lives.

These experiences are not unique to the mental healthcare sector in Canada. For example, Black academics noted that white faculty found it difficult to believe that ABR existed in university spaces and were unable to identify microaggressions that were not overt [41]. Canada has historically adopted a 'colour-blind' approach, and ABR has not been fully acknowledged until recently. BSC encapsulates this occurrence within a Canadian context, noting that'white professionals strip experiences presented by Black women, and re-present partial or distorted accounts more palatable to white frames of analysis’ [39].

### Black women taking on multiple roles

In additional to experience racial and sexual harassment at work, study results showed that Black women were overly burdened with the responsibility for caring for Black youth in a system that is failing to meet their needs [1]. ABR in the mental healthcare system is a systemic and organizational challenge that is being largely managed by individual practitioners [1]. Participants reported having to fill in the leadership gap at their places of employment to guarantee that Black youth could receive the care they deserved. To ensure young Black clients’ safety, respondents often took on multiple roles within their workplaces.

These findings align with the experiences of Black women in other contexts, who believed they alone could 'hold space' for their clients [42]. Participants’ personal experiences and community roles as caregivers significantly influenced their behaviour in the workplace as they felt responsible to ensure the safety of Black clients in their care because they understood the negative impact of ABR [43]. Black women's social location as both Black and women informs their feelings of responsibility for Black youth.

At the time of this study, Black communities in the US and Canada were grappling with the disproportionate number of deaths of Black people due to COVID-19 and the realities of the racial reckoning after the murder of George Floyd [10]. In many ways, participants were navigating similar experiences as their Black clients while also contending with the professional ramifications of addressing ABR within their workplaces, an experience previously echoed by Black mental health practitioners in the US [44]. These findings were similar to those of Lipscomb and Ashley [42], who noted that Black clinicians faced challenges and often felt the weight of the dual pandemic of ABR and COVID-19.

Despite this additional labour, Black women were rarely rewarded with career advancement or additional responsibilities that translated to a higher employment status [45]. Many Black women in this sample experienced of loneliness and isolation in these roles because the industry fails to hire enough Black women [1], despite placing a high value on their ability to do emotionally taxing work [12]. These findings were similar to those found by Holder et al. [46] who noted that Black women in leadership positions were often hypervisible because they were the ‘only one’ in their organizations and underrepresented. Similar findings occurred in Canada, as Premju and Etowa [47] found that Black nurses in Canada were hired to leadership positions less often compared to their counterparts.

### Impact on BWMH workers

The experiences of BWMH workers in this study are concerning as the effect of racism in mental healthcare workplaces has yet to be addressed in the Canadian context. Often the burden to provide solution falls on Black workers experiencing discrimination, as they are expected to navigate an uncooperative workplace environment. Participants stressed that there was a need for mental health organizations to address the anti-Black racism and sexism [8] directed at BWMH workers.

Unfortunately, BWMH were often penalized professionally for speaking up about their experiences of racialized sexual harassment which often affected their career trajectories. When BWMH workers reported discrimination, they were punished for highlighting the very systems of oppression that they were tasked with addressing. BWMH reported that their experiences were frequently minimized or ignored, which signalled to them that their concerns were unimportant.

Furthermore, the social, mental, and professional costs of stepping into the 'leadership gap' for Black service providers was often overlooked [42]. Participants expressed chronic stress, exhaustion, and burnout due to the ABR they experienced, negative effects of the Covid-19 pandemic, and the lack of support they received from their organizations, namely management and colleagues. By having to play the role of advocate in the workplace, BWMH workers were also forced to reinforce the 'strong Black woman' schema [46] which took a toll over time.

Respondents described how their frustrations increased when they realized that significant changes would not occur within their organizations despite their additional labour and efforts. For example, solutions were relegated to DEI checklists, with little changes made to organizational policies and practices. BWMH workers had a vested interest in these changes beyond improving care for Black youth, as many attempted to institute changes that would also improve their working conditions. However, conversations about race were eventually dissuaded or disregarded [12]. This is explicitly rooted in the Canadian context, as up until recently, conversations about race, racism and white supremacy were dissuaded mainly in favour of ignoring race altogether [48]. Thus, the experiences and efforts of Black women were further invisibilized. Similarly, discussions of race mirrored those occurring in the UK, where racism is largely ignored, and accusations are returned to Black women with 'proverbial chips on the shoulder' [12].

Black women also found themselves in a catch-22 when identifying solutions to address ABR, even if they were pushed to take on this additional labour. Respondents described experiencing pushback from their peers, often women, when attempting to make changes. For many respondents, their efforts were seen as a disruption to the status quo of their organizations, not unlike the challenges faced by their counterparts in the UK [12]. It is of note that many of these negative interactions took place in the presence of other non-Black women. These experiences catalyzed BWMH workers to leave the profession, which ultimately meant less representation and less culturally sensitive care for Black youth who sought care at their mental health organizations.

### Impact on the profession

In this article, participants often described leaving mental healthcare organizations to protect their mental health and wellbeing. The main reasons women left the profession or started their own practice were burnout, ABR, vicarious trauma, and lack of support. However, when BWMH workers found support and community they showed a renewed dedication to providing culturally appropriate care to youth and are breaking away from traditional mental health practices as a form of resistance.

As previously mentioned, study participants emphasized the presence of discrimination within the system, pointing to structural issues like unfair recruitment practices, a lack of BWMH workers hired within organizations and poor career progression. The systemic nature of this discrimination created numerous barriers for Black women in the workforce, discouraging them and negatively impacting worker retention [44, 45].

For BWMH workers in this study, burnout was attributed to the multitude of challenges that Black professionals face, including caring for others, responding to social crises, and trying to balance their personal needs. These findings align with extant research on mental health workers’ experiences after the Spring 2020 which revealed heightened feelings of being overwhelmed due to the multiple roles they had to undertake and the disproportionate burden of responsibilities they needed to manage [42, 44].

Ultimately, the experience of Black women in the mental healthcare workforce in Canada is one of increased emotional and professional labour, contending with stereotypes that threaten their career progression [49], and an undervaluing of their work and labour [50]. These realities, which are similar in the US [51] ultimately contribute to burnout and the desire to leave their roles in mainstream organizations to increase their ability to control their working environment—ultimately rejecting the need to be in white-dominated spaces [12]. However, the subsequent ramifications have consequences for the care Black youth receive within mainstream organizations and the profession.

#### Finding and giving support as a form of resistance

Having a supportive workplace proved vital for the research participants and played a crucial role in protecting the wellbeing of BWMH workers and positively impacted the quality of care for Black youth receiving mental health services. Thus, creating an environment that acknowledges both the collective and individual experiences of Black women, recognizing both commonalities and differences, is essential for fostering support among Black mental health workers. Research by Cupid & Bogues [50] highlighted that support from other Black women, through activities like sister circles emerged as a beneficial practice to address this burden, providing a space for validating experiences, connecting with like-minded individuals, and indirectly offering mental health support. A similar practice in workplaces may serve to increase feelings of belonging among BWMH workers and increase their ability to enact change by leveraging their community to drive collective action.

On a positive note, BWMH workers who left and established or joined private practices found solidarity and empowerment within their profession. These professionals created collective spaces for stigmatized and marginalized groups, addressing social inequalities, challenges, and systemic barriers affecting Black individuals and communities. This aligns with studies by Cupid & Bogues [50] and Goode-Cross and Grim [52] which noted that when Black workers experience solidarity in their workplaces, there was an increased commitment to addressing the systemic needs of Black clients and achieving better health outcomes.

Many BWMH workers tackled social justice and inequality by diverging from mainstream mental health care. They shifted towards providing care centred on lived experiences, racism, and Afrocentric principles. Afuape [53] advocates for a solidarity practice that resists mainstream psychology, psychiatry, and social policy through humility and social justice, contrary to mainstream services relying on neutrality and expertise.

In line with ASGB and BSC, the concept of African Sista-Hood, Black womanhood, and representation was a recurring theme among the participants [39]. Recognizing the diverse identities of individuals and the importance of seeing oneself represented in the workplace was crucial for both mental healthcare workers and the young people receiving services. Participants stressed the need for more Black leadership in workplaces, emphasizing that leadership should reflect the communities it serves. Goode-Cross and Grim [52] discovered that having Black individuals in higher leadership roles increased commitment to serving Black clients and the community. Furthermore, to foster effective therapeutic relationships, institutions must prioritize creating environments where staff and youth feel better represented by having more personnel who resemble the communities they serve.

### Implications

The results of this article contribute to the extant literature by demonstrating that the systemic nature of ABR works similarly across nation states and has changed very little in recent decades. Tuffour [45] explored the experiences Black African mental health nurses in the UK and found that they were often subject to discrimination and penalization due to their race, nationality, and primary language. Similarly, nearly 30 years ago Calliste [29] identified that nurses in Canada experienced differential treatment, were prevented from obtaining leadership positions and were more likely to be terminated. BSC draws on Black feminist theories illustrating that historical racism against Black people persists to this day and continues to impact their employment experiences. Thus, there is a need for more concerted efforts to address systemic ABR for BWMH workers.

The Canadian focus on BSC and African Sistahood draws linkages from the experiences of Black women in the UK, where women are faced with similar experiences of isolation. Moreover, an intersectional lens allows one to critique how both Black [54] and feminized labour are devalued in Canada [40]. Intersectionality and BCS provides us with a lens through which to view the experiences of BWMH workers who feel obliged to do this additional work, and address the organizational and institutional failures that fail to meet the needs of Black youth [1]. As participants challenge the systems of racism that affect Black youth, their experiences in the workplace are simultaneously informed by the racism and sexism that places them in this position [11].

Intersectionality began as a critique of white feminism [15], and is rooted in an understanding that Black women were not always fully included within it. Indeed, in this instance, non-Black women may have been reluctant to commit to *real* transformational change, instead attempting to maintain a 'normalcy' around race [55], a reality that is reminiscent of the call to action by the Combahee River Collective who specifically noted in their call Black women's devalued status in the labour force [56].

As it relates to stereotyping in the workplace, ASGB and therefore BSC recognizes the tension between hypervisibility and invisibility in the workplace [39] and notes that Black workers are simultaneously hypervisible, meaning they are often the focus of negative stereotypes and oversurveillance, and invisible because their challenges are overlooked and they lack the power to enact change. Indeed, the dialectic between hypervisibility and invisibility is socially constructed to maintain differential power structures within the workplace [40]. When Black women became impromptu leaders, they often became hypervisible within their organizations [12], and described feeling like 'mascots' for the organizations who upheld them and their work as examples of organizations' commitment to transformational change related to ABR. While participants often encouraged the commitment to changes, this position was also challenging because they often enacted these changes alone, felt the additional burden of making institutional changes with little support, and, unfortunately, were often punished career-wise for disrupting the status quo. At the same time, the additional work that Black women undertook was rarely compensated, contributing to the phenomenon of Black women being overworked and underpaid [50].

To address the challenges BWMH workers face, leadership at mental health organizations can implement policies and procedures that target microaggressions, inequitable recruitment and promotion, burnout, and the lack of trauma-informed mental health, and inequitable hiring and promotions. To sustain improvements to the experiences of BWMH workers, organizational leadership need to demonstrate commitment to lead efforts to mitigate ABR at their companies and remain accountable to missteps. Making organizational changes that improve Black representation, provide mental health support for BWMH workers, and equitable compensation can improve retention rates and the quality of care they can provide to Black youth [57].

### Recommendations

#### ABR, Unfair recruitment, and microaggressions

- Scrutinize workplace recruitment processes to identify and address potential biases against Black women.
- Advocate for mandated policies that target systemic barriers and promote equality for people of colour.
- Implement workplace audits to ensure that these policies are actively enforced.
- Organizations should provide ongoing mandatory education about the systemic barriers faced by Black women workers.

#### Burnout

- BWMH workers should regularly self-reflect, asking themselves whether they are stretching too thin to support their community.
- Emphasize self-care and prioritizing a healthy work-life balance is crucial.
- Paid mental health leave for professionals in the healthcare sector.

#### Support

- Organizations should cultivate supportive workplaces where Black mental health workers can openly discuss challenges and individual needs.
- Empowering women to voice their struggles and feelings of being unsupported is crucial.
- Advocate for better pay and workplace benefits.
- Provide culturally sensitive mentors to support new workers.
- Invest in workplace support training.

#### Vicarious trauma

- Mental healthcare workers must remain vigilant in understanding and maintaining boundaries with clients to minimize the risk of vicarious racial trauma.
- Workplaces should offer various resources on vicarious trauma and ABR.
- Workplaces should facilitate open discussions like debriefs and provide culturally responsive counselling to employees.

#### Leaving the profession

- Organizational efforts should focus on fostering a workplace culture of collectivity and solidarity for Black workers.
- Promoting Black individuals to leadership roles to shape workplace policies and practices nuanced to the Black diaspora.

#### Support as a form of resistance

Schools should enhance education on the nuances of Black health, moving away from applying mainstream mental health services to Black individuals and communities.

#### Black representation

Address the underrepresentation of BWMH workers at various levels—systemic, organizational, and individual—to ensure that young people not only receive effective mental healthcare, but also achieve the best possible health outcomes.

### Limitations

This article explores the experiences of BWMH workers and how those experiences affect both the profession and Black youth. While we extensive data on was collected BWMH workers’ influence on the profession, there is minimal data focused on how they impact Black youth directly. Engaging in intimate conversations with Black youth using mental health services and exploring the influence of BWMH workers on their journey could provide additional insight and a more nuanced understanding of the mental health landscape in Ontario.

This gap in knowledge suggests a need for future research that prioritizes the voices of Black youth receiving mental health care. Additional studies could explore the crucial role BWMH workers played in shaping these experiences. Recognizing the importance of Black mentors and therapists, a closer look at how these relationships positively affect the mental health profession and the wellbeing of Black youth could provide valuable insights for future research and policies. Further research on the workplace experiences of BWMH workers across all settings, including hospitals is needed as this study was mainly focused on mental healthcare organizations. A deeper understanding of the challenges BWMH workers experience and the subsequent impact on clients could drive critical long-term changes needed in the sector.

## Conclusion

This paper aimed to highlight and document the experiences of BWMH workers in Canada. Using the lens of a new theory, BSC, findings suggest that Black women experience discrimination related to ABR and sexism in the mental healthcare workforce and that they are often put into positions that limit their ability to experience a safe working environment and attain professional success. These findings also have implications for the care provided to Black youth seeking mental health support. By amplifying the voices of Black women, this article illustrates that there is a link between the experiences of Black mental healthcare professionals and the care provided to Black youth. A mental healthcare system that is harmful to BWMH workers will face difficulty adequately caring for Black youth. There is a clear need for systems-wide interventions related to discrimination within the workplace for these professionals. Organizational leaders and policymakers must acknowledge and address the anti-Black sexism and ABR that exist within the Canadian mental health care system and take meaningful action to address and eliminate discrimination directed toward this population. Care equity for Black youth can only be achieved when there is equity in the workplace for the providers that serve them.

## Supplementary Information

Supplementary Material 1.

## Data Availability

The datasets used and/or analysed during the current study are available from the corresponding author on reasonable request.

## Footnotes

1 Professor Akua Benjamin defines anti-Black racism as the “policies and practices which are rooted in Canadian institutions such as education, health care, and justice that mirror and reinforce beliefs, attitudes, prejudice, stereotyping and/or discrimination towards people of Black-African descent” (Black Health Alliance). Anonymous (2022) [57].

## References

[1] Fante-Coleman T, Jackson-Best F, Booker M, Worku F. Organizational and practitioner challenges to Black youth accessing mental health care in Canada: Problems and solutions. Can Psychol Psychol Can. 2023; Available from: http://doi.apa.org/getdoi.cfm?doi=10.1037/cap0000370. Cited 31 Jul 2023.

[2] Salami B, Denga B, Taylor R, Ajayi N, Jackson M, Asefaw M, et al. Original qualitative research - Access to mental health for Black youths in Alberta. Health Promot Chronic Dis Prev Can Res Policy Pract. 2021;41(9):245–53 Available from: https://www.ncbi.nlm.nih.gov/pmc/articles/PMC8565491/. Cited 12 Feb 2024. 10.24095/hpcdp.41.9.01 PMC8565491 34549916

[3] Fante-Coleman T, Jackson-Best F. Barriers and Facilitators to Accessing Mental Healthcare in Canada for Black Youth: A Scoping Review. Adolesc Res Rev. 2020;5(2):115–36. Available from: 10.1007/s40894-020-00133-2.

[4] Hassen N, Lofters A, Michael S, Mall A, Pinto AD, Rackal J. Implementing Anti-Racism Interventions in Healthcare Settings: A Scoping Review. Int J Environ Res Public Health. 2021;18(6):2993 Available from: https://www.mdpi.com/1660-4601/18/6/2993. 33803942 10.3390/ijerph18062993 PMC8000324

[5] Faber SC, Khanna Roy A, Michaels TI, Williams MT. The weaponization of medicine: Early psychosis in the Black community and the need for racially informed mental healthcare. Front Psychiatry. 2023;14:1098292. Available from: https://www.frontiersin.org/articles/10.3389/fpsyt.2023.1098292/full. Cited 13 Jun 2023. 36846217 10.3389/fpsyt.2023.1098292 PMC9947477

[6] Anglin DM, Ereshefsky S, Klaunig MJ, Bridgwater MA, Niendam TA, Ellman LM, et al. From Womb to Neighborhood: A Racial Analysis of Social Determinants of Psychosis in the United States. Am J Psychiatry. 2021;178(7):599–610. Available from: https://ajp.psychiatryonline.org/doi/full/10.1176/appi.ajp.2020.20071091. Cited 25 Jul 2023 33934608 10.1176/appi.ajp.2020.20071091 PMC8655820

[7] Cénat JM, Dromer É, Darius WP, Dalexis RD, Furyk SE, Poisson H, et al. Incidence, Racial Disparities and Factors Related to Psychosis among Black Individuals in Canada: A Scoping Review. Can J Psychiatry. 2023;07067437231178957. Available from: 10.1177/07067437231178957. Cited 1 Aug 2023. 10.1177/07067437231178957 PMC10517652 37269120

[8] Noble D, Palmer LA. Misogynoir: Anti-Blackness, Patriarchy, and Refusing the Wrongness of Black Women. In: Tate SA, Gutiérrez Rodríguez E, editors. The Palgrave Handbook of Critical Race and Gender. Cham: Springer International Publishing; 2022. p. 227–45. Available from: 10.1007/978-3-030-83947-5_12.

[9] Office of the Auditor General of Ontario. Performance Audit: Community-Based Child and Youth Mental Health Program. Province of Ontario; 2025. Available from: https://auditor.on.ca/en/content/annualreports/arreports/en25/pa_CYMH_en25.pdf.

[10] Canadian Nurses Association. Anti-Black racism is a public health emergency in Canada. 2020. Available from: https://www.cna-aiic.ca/en/blogs/cn-content/2020/06/11/anti-black-racism-is-a-public-health-emergency-in.

[11] Crenshaw K, Creer J, Cheung E, Rimonte N, Smith F, Banks T, et al. Mapping the Margins : Intersectionality, Identity Politics, and Violence Against Women of Color Mapping the Margins : Intersectionality, Identity Politics, and Violence Against Women of Color. Stanford Law Rev. 1991;43(6):1241–99.

[12] Obasi C. Black social workers: Identity, racism, invisibility/hypervisibility at work. J Soc Work. 2022;22(2):479–97. Available from: 10.1177/14680173211008110. Cited 28 Nov 2023.

[13] Booker M, Jackson-Best F, Fante-Coleman T. A Social Network Analysis of Interagency Collaboration in the Mental Health Sector in Toronto, Canada: Service Providers’ Perspectives on Supporting Black Youth in Recovery. J Recovery Ment Health. 2023;6(2):4–32 Available from: https://jps.library.utoronto.ca/index.php/rmh/article/view/39734. Cited 31 Jul 2023.

[14] Fante-Coleman T, Allen K, Booker M, Craigg A, Jackson-Best F. “If You Prayed More, You Would Feel Better”: The Dual Nature of Religion and Spirituality on Black Youths’ Mental Health and Access to Care in Canada. Child Adolesc Soc Work J. 2023. Available from: 10.1007/s10560-023-00932-1.

[15] Collins PH. Intersectionality’s Definitional Dilemmas. Annu Rev Sociol. 2015;41(1):1–20. Available from: https://www.annualreviews.org/doi/10.1146/annurev-soc-073014-112142.

[16] Saroukhani H, McLeod J. A Loss of Innocence. Wasafiri. 2023;38(2):1–3. Available from: 10.1080/02690055.2023.2171610. Cited 7 Aug 2024.

[17] Crenshaw K, editor. Critical race theory: the key writings that formed the movement. New York: New Press; 1995. p. 494.

[18] Derworiz C. “One of the biggest Black settlements in Western Canada” has a rich history | CBC News. CBC. 2021. Available from: https://www.cbc.ca/news/canada/edmonton/black-settlement-alberta-amber-valley-1.5900836 . Cited 15 Dec 2023.

[19] Robb N. Racism can rear its ugly head at medical school, study finds. CMAJ. 1998;159(1):66–7. 9679492 PMC1229488

[20] Wilson C. Racial profiling, street checks continue despite elimination of “carding,” report on Toronto police finds. CP24. 2023. Available from: https://www.cp24.com/news/black-torontonians-continue-to-face-racial-profiling-street-checks-despite-elimination-of-carding-report-finds-1.6687136. Cited 15 Dec 2023.

[21] Marshall EZ. Introduction: Power, Performance and Play: Caribbean Carnival and the Cultural Politics of Emancipation. Caribb Q. 2019;65(4):483–90. Available from: https://www.tandfonline.com/doi/full/10.1080/00086495.2019.1682345. Cited 7 Aug 2024.

[22] Peyson M. Divergence / convergence: caribbean diaspora as case study for advancing inclusive community engagement. 2024. Available from: http://hdl.handle.net/1993/38154. Cited 7 Aug 2024.

[23] Walker JWStG. Diaspora Dynamics: Canadian Immigration Policy and the Caribbean Connection. Hist Soc Soc Hist. 2022;55(114):249–70. Available from: https://muse-jhu-edu.myaccess.library.utoronto.ca/pub/273/article/872811. Cited 7 Aug 2024.

[24] Braun V, Clarke V. Using thematic analysis in psychology. Qual Res Psychol. 2006;3(2):77–101. Available from: http://www.tandfonline.com/doi/abs/10.1191/1478088706qp063oa. Cited 19 Jun 2023.

[25] Eakin JM, Gladstone B. “Value-adding” Analysis: Doing More With Qualitative Data. Int J Qual Methods. 2020;19:1609406920949333. Available from: 10.1177/1609406920949333. Cited 9 Aug 2023.

[26] Tracy SJ. Qualitative Quality: Eight “Big-Tent” Criteria for Excellent Qualitative Research. Qual Inq. 2010;16(10):837–51. Available from: http://journals.sagepub.com/doi/10.1177/1077800410383121. Cited 5 Nov 2023.

[27] Israel BA, Coombe CM, Cheezum RR, Schulz AJ, Mcgranaghan RJ, Lichtenstein R, et al. Community-based participatory research: a capacity-building approach for policy advocacy aimed at eliminating health disparities. Public Health. 2010;100:2094–102. 10.2105/AJPH.2009.170506 PMC2951933 20864728

[28] Feminist Methodology: New Applications in the Academy and Public Policy.. Available from: https://www.journals.uchicago.edu/doi/epdf/10.1086/428417. Cited 10 Jun 2025.

[29] Calliste A. Antiracism organizing and resistance in nursing: African Canadian women. Can Rev Sociol Can Sociol. 1996;33(3):361–90. Available from: http://go.galegroup.com.libproxy.wlu.ca/ps/retrieve.do?tabID=T002&resultListType=RESULT_LIST&searchResultsType=SingleTab&searchType=AdvancedSearchForm&currentPosition=1&docId=GALE%7CA19139742&docType=Article&sort=RELEVANCE&contentSegment=&prodId=AONE&cont . Cited 29 Jan 2018.

[30] Hsuing, Ping-Chung. Lives and Legacies. 2010. Reflexivity - A Process of Reflection. Available from: http://www.utsc.utoronto.ca/~pchsiung/LAL/reflexivity. Cited 7 Feb 2017.

[31] Fante-Coleman, T, Booker, M., Craigg, A, Plummer, D, Jackson-Best, F. Factors that Impact How Black Youth Access the Mental Healthcare System in Ontario. Toronto, ON: Black Health Alliance; 2022. Available from: https://www.pathwaystocare.ca/research/ptc-focus-group-report-eng. Cited 2 Feb 2024.

[32] Etikan I, Musa SA, Alkassim RS. Comparison of Convenience Sampling and Purposive Sampling. Am J Theor Appl Stat. 2016;5(1):1. Available from: http://www.sciencepublishinggroup.com/journal/paperinfo?journalid=146&doi=10.11648/j.ajtas.20160501.11. Cited 21 Jun 2023.

[33] Government of Canada IAP on RE. Tri-Council Policy Statement: Ethical Conduct for Research Involving Humans – TCPS 2 (2022). 2023. Available from: https://ethics.gc.ca/eng/policy-politique_tcps2-eptc2_2022.html. Cited 15 Jan 2024.

[34] Collins PH. Learning from the Outsider Within: The Sociological Significance of Black Feminist Thought. Soc Probl. 1986;33(6). Available from: https://academic.oup.com/socpro/article/33/6/s14/1610242.

[35] Center for Addications and Mental Health (CAMH). CAMH. 2024. Career Burnout. Available from: https://www.camh.ca/en/camh-news-and-stories/career-burnout. Cited 28 Oct 2024.

[36] Fante-Coleman T, Jackson-Best F. Barriers and Facilitators to Accessing Mental Healthcare for Black Children & Youth: A Scoping Review. 2020;30. Available from: https://www.researchgate.net/publication/342530665_Barriers_and_Facilitators_to_Accessing_Mental_Healthcare_for_Black_Children_Youth_A_Scoping_Review/citations.

[37] Anglin DM, Lui F, Espinosa A, Tikhonov AA, Ellman L. Ethnic identity, racial discrimination and attenuated psychotic symptoms in an urban population of emerging adults. Early Interv Psychiatry. 2018;12(3):380–90. Available from: https://onlinelibrary.wiley.com/doi/abs/10.1111/eip.12314. 26818635 10.1111/eip.12314

[38] Henry, F., James, C. E., Li, P. S., Kobayashi, A., Smith, M. S., Ramos, H., & Enakshi, D. (2017). The equity myth: Racialization and indigeneity at Canadian universities. UBC Press. https://www.ubcpress.ca/asset/20223/1/9780774834889

[39] Obasi, C. (2019). Africanist sista-hood in Britain: creating our own pathways. In A. Emejulu, & F. Sobande (Eds.), To Exist Is to Resist: Black Feminism in Europe Pluto Press.

[40] Buchanan NT, Settles IH. Managing (in)visibility and hypervisibility in the workplace. J Vocat Behav. 2019;113:1–5. Available from: https://linkinghub.elsevier.com/retrieve/pii/S0001879118301313. Cited 11 Oct 2024.

[41] Henry F, Tator C. Interviews with Racialized Faculty Members in Canadian Universities. Can Ethn Stud. 2012;44(1):75–99. Available from: https://muse.jhu.edu/article/509923. Cited 13 Jun 2025.

[42] Lipscomb AE, Ashley W. Surviving being Black and a clinician during a dual pandemic: Personal and professional challenges in a disease and racial crisis. Smith Coll Stud Soc Work. 2020;90(4):221–36.

[43] Hogan VK, de Araujo EM, Caldwell KL, Gonzalez-Nahm SN, Black KZ. “We black women have to kill a lion everyday”: An intersectional analysis of racism and social determinants of health in Brazil. Soc Sci Med. 2018;199:96–105. Available from: 10.1016/j.socscimed.2017.07.008. Cited 25 Sep 2018. 28760333 10.1016/j.socscimed.2017.07.008

[44] Miu AS, Moore JR. Behind the Masks: Experiences of Mental Health Practitioners of Color During the COVID-19 Pandemic. Acad Psychiatry. 2021;45(5):539–44. Available from: 10.1007/s40596-021-01427-w. Cited 28 Nov 2023. 33660237 10.1007/s40596-021-01427-w PMC7929548

[45] Tuffour I. It is like ‘judging a book by its cover’: An exploration of the lived experiences of Black African mental health nurses in England. Nurs Inq. 2022;29(1):e12436. Available from: https://onlinelibrary.wiley.com/doi/abs/10.1111/nin.12436. Cited 28 Nov 2023. 34124816 10.1111/nin.12436

[46] Holder A, Jackson MA, Ponterotto JG. Experiences of racial microaggressions and coping strategies of Black women in corporate America. Qual Psychol. 2015;2(2):164–80.

[47] Premji S, Spasevski M, Athar S, Coordinator R, Shakya Y, Merolli J, et al. Precarious Work Experiences of Racialized Immigrant Women in Toronto: a Community- Based Study. Just Labour Can J Work Soc. 2014;22:122–43.

[48] Mullings DV, Morgan A, KereQuelleng H. Canada the great white north where anti-black racism thrives: kicking down the doors and exposing the realities. Phylon Clark Atlanta Univ Rev Race Cult. 2016;1(53):20–41.

[49] Reynolds-Dobbs W, Thomas KM, Harrison MS. From mammy to superwoman: Images that hinder Black women’s career development. J Career Dev. 2008;35(2):129–50.

[50] Cupid S, Bogues K. “No filters needed .” A qualitative study exploring sister circles and workplace messages for Black women healthcare professionals during the double pandemic. Womens Health. 2023;19:17455057231181017. Available from: https://journals.sagepub.com/doi/10.1177/17455057231181017. Cited 22 Oct 2024. 10.1177/17455057231181017 PMC10293866 37358214

[51] Shell EM, Teodorescu D, Williams LD. Investigating Race-related Stress, Burnout, and Secondary Traumatic Stress for Black Mental Health Therapists. J Black Psychol. 2021;47(8):669–94. Available from: 10.1177/00957984211033963. Cited 28 Nov 2023.

[52] Goode-Cross DT, Grim KA. “An Unspoken Level of Comfort”: Black Therapists’ Experiences Working With Black Clients. J Black Psychol. 2016;42(1):29–53. Available from: 10.1177/0095798414552103. Cited 23 Oct 2024.

[53] Afuape T. Beyond awareness of ‘difference’ and towards social action: ‘Solidarity practice’ alongside young people. Clin Child Psychol Psychiatry. 2016;21(3):402–15. Available from: 10.1177/1359104516645642. Cited 22 Oct 2024. 27233811 10.1177/1359104516645642

[54] Probst TM, Sinclair RR, Sears LE, Gailey NJ, Black KJ, Cheung JH. Economic stress and well-being: does population health context matter? J Appl Psychol. 2018;103(9):959–79. 29733623 10.1037/apl0000309

[55] Feminist legal theories. Available from: https://books-scholarsportal-info.myaccess.library.utoronto.ca/en/read?id=/ebooks/ebooks4/taylorandfrancis4/2018-06-04/4/9781135634629#page=281. Cited 28 Nov 2023.

[56] Combahee River Collective. (2017). The Combahee River collective statement In Taylor, K-Y (Ed.), How we get free (pp. 15-27). Haymarket Books. https://www.reed.edu/cres/assets/Combahee-River-Collective,-Black-Feminist-Statement,-How-We-Get-Free---Taylor.pdf

[57] Black Health Alliance. Black Health Alliance. 2024. Anti-Black Racism. Available from: https://blackhealthalliance.ca/home/antiblack-racism/. Cited 5 Nov 2024.

