# Supplementary material for: Seeing both sides: detailing the experiences of Black women mental healthcare workers serving Black youth
Source: BMC Health Serv Res. 2025 Oct 10;25:1352. doi: 10.1186/s12913-025-13250-2 (PMC12512575; doi:10.1186/s12913-025-13250-2)
Supplement: Supplementary file 1 — Supplementary Material 1. [file 12913_2025_13250_MOESM1_ESM.docx]

**Focus Group Guide – Pathways to Care Project**

**Target Audience: Service Providers, Health Professionals and Decision-Makers**

**Stated Purpose:**

1. **To assess how organizations either help or hinder mental healthcare provision**
2. **To identify how anti-racism praxis is performed in the mental healthcare setting**
3. **To assess practitioners’ opinions on how best to provide care for Black youth**
4. **Impact the Strategic Framework – What do youth want to see from Policy Makers? Organizations? Providers? What can do to make it easier to access care?**

***Hi everyone, my name is ______ and my pronouns are _________. and I’d like to welcome and thank you for participating in this focus group. We will be asking some questions about mental health for Black Children, and youth and access to mental healthcare. Your responses will help the Pathways to Care project and its stakeholders to make recommendations for policymakers, mental health organizations and service practitioners. It will also help the Pathways to Care project to create partnerships with mental health care organizations that want to serve Black children and youth.***

***Our discussion today will take about 90 minutes and we’ll take some breaks along the way to stretch our legs. This conversation is informal so please speak freely when answering questions and feel free to respond to other participants’ comments. If you don’t understand a question, please let me know. I am here to ask questions, listen, and make sure everyone has a chance to share. Please know that you are not obligated to answer any question, you can always choose to say you ‘pass,’ no questions asked.***

***We do have a limited time together today, so if we start to get off topic, I may interrupt you so we can move forward. Don’t feel bad about it, group conversations often get sidetracked since we all want to share.***

***We will be tape recording the discussion tonight because we don’t want to miss any of your comments. No one outside of our research team will have access to the data. No names will be included in any report and your comments are confidential.***

***We also request that you make sure personal comments don’t leave the room. I hope you’ll feel free to speak openly and honestly. We will be providing resources at the end of our discussion. For any reason if you have to leave please give us a ‘thumbs-up’ if you are ok, and a ‘thumbs-down’ if you would like some assistance.***

***I am starting the Audio Recorder now.***

**Introduction Questions**

1. **To begin, why don’t we all introduce ourselves and where you live [city]?**
2. **Next, why don’t we discuss how you came to be in this focus group? What are your expectations for this meeting?**

**Organizational Questions**

1. **How would you describe your organization's practice focus?**
   1. **PROMPT What is the practice approach?**
2. **How would you describe the support you receive [in general] from the organization that you work for?**
3. **Are any equity policies/practices incorporated into the practices at your workplace? If so, how?**
4. **What are your thoughts on interdisciplinary teams in mental healthcare?**

**Practice Questions**

1. **For your clients, what is the most salient barrier to care for you?**
   1. **PROMPT Financial?**
   2. **PROMPT Geographical?**
   3. **PROMPT Stigma?**
2. **What is the practice model at your workplace?**
   1. **PROMPT What is the practice approach?**
   2. **PROMPT What do the working assumptions and/or policies look like?**
3. **Have these practice models been assessed for cultural competence?**
4. **How would you assess if someone had a mental health ‘problem’?**
5. **Does ‘race’ play a role in assessing whether someone has a mental health ‘problem’?**
   1. **PROMPT Do the assessment tools you use to make an assessment/diagnosis factor-in ‘race’?**
   2. **PROMPT Does it include experiences of racism/race-based discrimination?**
6. **In what way do you factor the role of racism/race-based discrimination in a client’s mental health into your practice/service provision?**
7. **In other focus groups, we are having conversations with Black youth who identify as 2SLGBTQ+ and those who have experience in the justice system. In your experience what are the unique barriers for these groups, respectively?**
8. **Describe how intergenerational trauma is addressed by your organizational practices and/or treatment practices**
9. **What are your biggest challenges in working with/providing care to Black youth?**
   1. **What would you change about the work you are currently doing?**

**Envisioning a Better Practice**

1. **What information do you think is currently lacking concerning mental health provision for Black youth?**
2. **What treatment practice do you believe needs more attention to make sure it is evidence-based?**
3. **If you have done so, describe some of the ways that you have innovated providing care within the constraints of current standard practice?**
4. **What tools could be provided to help you better serve Black children and youth and their families?**
5. **What changes could be made to organizational practices to make serving Black children, youth and their families easier?**
6. **Previous research that we’ve done has shown that there’s a disconnect between anti-racist theory and practice. What does anti-racist practice actually look like to you?**
   1. **PROMPT: How can we embody anti-racist praxis in the room?**
7. **In other focus groups, we talked with youth who identify as 2SLGBTQ+ and youth who have experience in the justice system. How can intersectionality play an actual role in clinical treatment for these groups?**
8. **How can religion play an actual role in clinical treatment?**
9. **How can barriers related to the social determinants of health be addressed in making sure consumer survivors continuously receive care?**

***I am stopping the audio recorder now. Do you have any questions off the record?***

***Thank you so much for taking the time to share with us today. Your responses will help us to better serve Black children and youth seeking mental healthcare in Ontario. Your input today is so important and will impact the direction of the project greatly. We have a list of resources available to you if you would like them.***
